# A Few Old Simples

**Published:** 1889-11-30

**Authors:** 


### A Few Old Simples.

The present state of medical science as compared with that of a few centuries ago amply justifies the trite old saying, "*Tempora mutantur, nos et mutamur in illis*"—"The times are perpetually changing, and we with them." The simple and complex were then strangely mingled. Sometimes as many as thirty, or even forty, ingredients composed one nostrum of our forefathers in pharmacy, on the chance of one, at least, among the number hitting at the root of the disease.

But, to begin with, there was an infallible means of testing whether or no the patient was worth the thought and care of the physician, or, in other words, whether he would recover or no. Unwittingly, he was made to pronounce his own doom. "If the good friend were sick," it was customary for the doctor, when he took his hand and enquired what he thought of his condition, to secrete in his own right palm a leaf or two of the plant *vervain*, in such a manner that the patient "wot not of it." If, in his innocence, he replied that he felt sure that his indisposition was merely temporary, it was an augury that he would certainly respond to the remedies prescribed. If, on the contrary, however, he took a despondent view of his case, he would as certainly die. Magic properties were attributed to this species of the verbena by our ancestors. In Drayton's "Nymphidia" we read:

"She nightshade strows to work him ill,
Therewith the *vervain* and the dill,
That hind'reth witches of their will."

Let us hope and believe that the leech of the past was equally careful to hide from the sick man the compositions of the drugs he was made to swallow, and of the ointments prescribed for his external use, for, unless our ancestors were less fanciful in their tastes than ourselves, many of the remedies resorted to must have induced a mental and physical qualm. But fastidiousness and prejudice may, perhaps, be reckoned among the many vices of the nineteenth century. How would a victim of whooping-cough feel were he compelled, knowingly, to swallow a draught of ale, as medicine, with which a skinned mouse had been incorporated, dried in an oven, and "stamped" to powder? He would doubtless prefer to adopt the alternative of crawling three times before breakfast under a blackberry bramble, both ends of which were growing in the soil.

A decoction of earth-worms, English saffron, and scraped ivory effected "marvellous moche goode" in cases of jaundice, and epilepsy was subdued by one composed of moss which had grown on the skull of a person who had died a violent death, of polypode of oak well dried, parings of human nails, root of dried peony, and mistletoe, which must have been gathered during the wane of the moon. Another invaluable remedy for the same complaint, then known as the "falling sickness," was a plaster, applied before the fit came on, to the wrists and the soles of both feet, composed of snails pounded with their shells in a mortar, baysalt, and flowers of the mallow. But a certain magic word pronounced in the ear often of itself sufficed to prevent its recurrence. Again, twenty snails in the shells, reduced to a paste, was a common remedy for boils, while a drop or two of their juice, mixed with samphire, was good for a corn.

To allay hæmorrhage, a toad, well dried in the sun and put into a bag, was hung round the neck by a string sufficiently low to touch the region of the heart; and a preparation of garlic and honey smeared on the person was said to act as a charm against the bites of dogs and reptiles, or the sting of numerous insects, likewise effecting their cure. Toothache could be charmed away by a few leaves of the "shepherd's purse," placed in the sole of the shoe on the reverse side of the body to that in which the tooth was aching.

An excellent recipe for weak or sore eyes was the expressed juice of the calyx of the red honeysuckle; provided always that the flowers were gathered kneeling, repeating nine paternosters in honour of the Trinity, nine more "to greet Our Ladye," and a creed. Rest and sleep were required after application. Another prescription for the eyes much in favour with the Anglo-Saxons was a paste made of the strawberry-plant and pepper, diluted with sweet wine. Children were passed through the split stem of a tree for the cure of the rickets, but the fracture must be afterwards bound up sufficiently tightly to ensure cohesion. For ague a very well-salted herring, split open, was applied, as hot as as possible, to the soles of the feet. It might also be mitigated by the habitual wearing round the neck of an emerald—a gem equally potent in epilepsy.

Precious stones were accredited with marvellous powers over the moral qualities and affections, as well as physical disease—hence the origin of their being set in rings and worn. The diamond, though a deadly poison if swallowed, guaranteed the wearer from the fatal effects of any poisonous drug or wound. The ruby, possessed of similar virtue, was likewise a guarantee against plague, changing colour and turning dim in presence of danger, and again becoming bright when that danger was past. The onyx strengthened the heart morally and physically; the teeth of old age were fixed firmly in the gums by an infusion of powdered jet; while water in which the beryl had been steeped afforded a valuable wash for strengthening the eyes, besides ensuring the mutual love of a wedded couple.

Much latent humour is discernible in some of the recipes of our forefathers, as, for instance, that saffron, cloves, musk, balm, mirth and gladness are good for diseases of the heart, and that beans, peas, sadness, anger, onions, evil tidings, loss of friends are bad; that rue diminishes the force of love in man, but strengthens it in woman; that, when eaten raw, it clears the perceptions of the mind and eyes, destroying fleas when cooked; and that the human race is preserved by sage.

Unfortunately, the limits of space are less inexhaustible than is our subject, and these prescribe an abrupt halt in the motions of the goose-quill.

